# Supplementary material for: Integrating deep learning and unbiased automated high-content screening to identify complex disease signatures in human fibroblasts
Source: Nat Commun. 2022 Mar 25;13:1590. doi: 10.1038/s41467-022-28423-4 (PMC8956598; doi:10.1038/s41467-022-28423-4)
Supplement: Supplementary file 2 — Reporting Summary [file 41467_2022_28423_MOESM2_ESM.pdf]

nature research

Corresponding author(s): Bjarki Johannesson
Samuel Yang
Marc Berndl

Last updated by author(s): 12/13/2021

# Reporting Summary

Nature Research wishes to improve the reproducibility of the work that we publish. This form provides structure for consistency and transparency in reporting. For further information on Nature Research policies, see our Editorial Policies and the Editorial Policy Checklist.

## Statistics

For all statistical analyses, confirm that the following items are present in the figure legend, table legend, main text, or Methods section.

| n/a | Confirmed | |
|---|---|---|
| ☐ | ☒ | The exact sample size (*n*) for each experimental group/condition, given as a discrete number and unit of measurement |
| ☐ | ☒ | A statement on whether measurements were taken from distinct samples or whether the same sample was measured repeatedly |
| ☐ | ☒ | The statistical test(s) used AND whether they are one- or two-sided<br>*Only common tests should be described solely by name; describe more complex techniques in the Methods section.* |
| ☐ | ☒ | A description of all covariates tested |
| ☒ | ☐ | A description of any assumptions or corrections, such as tests of normality and adjustment for multiple comparisons |
| ☐ | ☒ | A full description of the statistical parameters including central tendency (e.g. means) or other basic estimates (e.g. regression coefficient) AND variation (e.g. standard deviation) or associated estimates of uncertainty (e.g. confidence intervals) |
| ☐ | ☒ | For null hypothesis testing, the test statistic (e.g. $F$, $t$, $r$) with confidence intervals, effect sizes, degrees of freedom and $P$ value noted<br>*Give P values as exact values whenever suitable.* |
| ☒ | ☐ | For Bayesian analysis, information on the choice of priors and Markov chain Monte Carlo settings |
| ☒ | ☐ | For hierarchical and complex designs, identification of the appropriate level for tests and full reporting of outcomes |
| ☐ | ☒ | Estimates of effect sizes (e.g. Cohen's *d*, Pearson's *r*), indicating how they were calculated |

*Our web collection on statistics for biologists contains articles on many of the points above.*

## Software and code

Policy information about availability of computer code

| | |
|---|---|
| Data collection | NIS-Elements AR image acquisition software (5.21.03 LO) was used to acquire the image dataset. The near real-time image quality analysis is available as a Fiji (an ImageJ distribution) macro at https://github.com/google/microscopeimagequality/tree/main/wellmontagefijimacro. |
| Data analysis | CellProfiler 3.1.5 was used to produce cell features. Code for generating a deep embedding from an image as well as fitting and evaluating the cell line and PD classification models is available at https://nyscf.org/nyscf-adpd/. |

For manuscripts utilizing custom algorithms or software that are central to the research but not yet described in published literature, software must be made available to editors and reviewers. We strongly encourage code deposition in a community repository (e.g. GitHub). See the Nature Research guidelines for submitting code & software for further information.

## Data

Policy information about availability of data

All manuscripts must include a data availability statement. This statement should provide the following information, where applicable:
- Accession codes, unique identifiers, or web links for publicly available datasets
- A list of figures that have associated raw data
- A description of any restrictions on data availability

The raw and pre-processed full-resolution images data are available under restricted access due to dataset size constraints, access can be obtained by a data request form can be found at https://nyscf.org/nyscf-adpd/. The processed image, deep embedding, and CellProfiler data are available under the CC BY-NC-SA 4.0 license at https://nyscf.org/nyscf-adpd/.

# Field-specific reporting

Please select the one below that is the best fit for your research. If you are not sure, read the appropriate sections before making your selection.

☒ Life sciences ☐ Behavioural & social sciences ☐ Ecological, evolutionary & environmental sciences

For a reference copy of the document with all sections, see nature.com/documents/nr-reporting-summary-flat.pdf

# Life sciences study design

All studies must disclose on these points even when the disclosure is negative.

**Sample size**

96 primary fibroblast lines from 91 individuals with Parkinson's disease and healthy controls, forming 45 demographically matched pairs were chosen from the New York Stem Cell Foundation Fibroblast Repository. Each line was profiled in 48 wells, each containing more than 1000 cells, resulting in a total of dataset of 48 terabytes.

**Data exclusions**

As detailed in the manuscript, we omitted GBA PD samples from the disease classification task because our analysis of these lines indicated a possible underlying stratification imbalance in our partitioning of GBA cell lines (Parker et al., 2007) across cross-validation datasets demonstrated by a majority of the splits achieving AUCs well below 0.5 (Supplementary Fig. 4). Interestingly, all of the PD donors in the study were patients at the same academic medical center in New York City except for three, including two of the 8 GBA patients, a potential, but unverified confound.

**Replication**

All 96 fibroblast lines were profiled in 4 independent batches where each batch consisted of two distinct plate layouts. A major goal of this work was to achieve technical reproducibility that would allow cross-batch validation. To this end, we adopted a cross-validation scheme where a model is fit to three of the four batches and its performance was evaluated on the fourth, held out batch. All model figures of merit numbers reported are from test sets. Importantly, we also held out the plate layout to ensure that the model was unable to rely on any possible location biases. High classification accuracy was achieved with this approach, showcasing the batch-to-batch reproducibility of our platform.

**Randomization**

Fibroblast lines were divided into experimental group based on the disease diagnosis of the donor, i.e. PD and healthy controls. Healthy controls were age-, sex- and ancestry-matched with PD counterparts. Plate layouts were populated at random with healthy-disease cell line pairs.

**Blinding**

Scientists that performed the automated expansion and freeze-down of fibroblast were blinded to experimental groupings. For each of the 4 identical profiling batches, cell thawing, adaptation, seeding into assay plates and Cell Painting was performed using a fully automated platform, eliminating the possibility of experimental bias stemming from manual procedures. Further, in each batch, two plate layouts were used, which alternated healthy and PD lines every other well and also positioned healthy and PD lines paired by both age and sex in adjacent wells, when possible.

# Reporting for specific materials, systems and methods

We require information from authors about some types of materials, experimental systems and methods used in many studies. Here, indicate whether each material, system or method listed is relevant to your study. If you are not sure if a list item applies to your research, read the appropriate section before selecting a response.

## Materials & experimental systems

| n/a | Involved in the study | |
|---|---|---|
| ☒ | ☐ | Antibodies |
| ☐ | ☒ | Eukaryotic cell lines |
| ☒ | ☐ | Palaeontology and archaeology |
| ☒ | ☐ | Animals and other organisms |
| ☐ | ☒ | Human research participants |
| ☒ | ☐ | Clinical data |
| ☒ | ☐ | Dual use research of concern |

## Methods

| n/a | Involved in the study | |
|---|---|---|
| ☒ | ☐ | ChIP-seq |
| ☒ | ☐ | Flow cytometry |
| ☒ | ☐ | MRI-based neuroimaging |

## Eukaryotic cell lines

Policy information about cell lines

**Cell line source(s)**

Primary skin fibroblasts used in this study were from the New York Stem Cell Foundation Fibroblast Repository. Fibroblasts were derived from skin biopsies collected at the same dermatology clinic and expanded using standardized, automated procedures using the New York Stem Cell Foundation Global Stem Cell Array®.

**Authentication**

DNA from newly derived fibroblast lines was collected and analyzed using Fluidigm SNPTrace. The same analysis was

| | |
|---|---|
| Authentication | performed following expansion of the lines profiled in this study, confirming their identity to be the same as the original lines. |
| Mycoplasma contamination | All cell lines used in the study were confirmed to be negative for mycoplasma contamination. |
| Commonly misidentified lines (See ICLAC register) | Only verified primary fibroblast lines were used in this study. |

## Human research participants

Policy information about studies involving human research participants

| | |
|---|---|
| Population characteristics | Primary fibroblasts from 91 individuals were used in this study, out of which 5 donated 2 biopsies (3-6 years apart) that were analyzed as individual lines. All lines were sourced from the same dermatology clinic and were processed and expanded using highly standardized, automated procedures using the New York Stem Cell Foundation Global Stem Cell Array(R). The 91 individuals included 32 sporadic PD, 8 GBA PD and 6 LRRK2 PD that were meticulously age-, sex- and ancestry-matched with 45 healthy controls. The human research participants consisted of 36 females and 60 males between the ages of 45 and 81, with the average age being 64. 48 participants were healthy, while 33 had Sporadic PD, 7 had the LRRK2 mutation and 8 had the GBA mutation. The demographic matching of cell lines is detailed in the manuscript. Importantly, we confirmed the robustness of our experimental design quantitatively with a detailed propensity score analysis of disease state as a function of plate and cell line covariates, which did not reveal any significant confounds. |
| Recruitment | Participants were recruited through a variety of means, including (but not limited to) physician referral, Fox Trial Finder, Craigslist, CenterWatch, and Clinicaltrials.gov. As participants self-select, there is a possibility that those who participated may be different in experience and/or exposure than those who did not. For instance, there may be some socioeconomical bias in individuals that opt to participate as this participation takes some time and effort, and it is conceivable that individuals that are unwell will be less likely to participate. Importantly, the demographics of PD subjects and healthy controls were carefully matched. Participants provided written informed consent. A $50 gift card was provided if the consent version used at the time of collection offered compensation. |
| Ethics oversight | Institutional Review Board |

Note that full information on the approval of the study protocol must also be provided in the manuscript.
