# Supplementary material for: Integrating deep learning and unbiased automated high-content screening to identify complex disease signatures in human fibroblasts
Source: Nat Commun. 2022 Mar 25;13:1590. doi: 10.1038/s41467-022-28423-4 (PMC8956598; doi:10.1038/s41467-022-28423-4)
Supplement: Supplementary file 3 — Description of Additional Supplementary Files [file 41467_2022_28423_MOESM3_ESM.pdf]

Description of Additional Supplementary Files

File Name: Schiff-et-al.-Supplementary-Data.xlsx, Supplementary Data 1

Description: Information about the 96 cell lines from 91 donors used in the study. Columns left to right: ID mapping to a cell line from a unique biopsy; the biopsy donor; the cross-validation fold for disease prediction; ID for paired PD healthy control; PD status; donor sex; donor age; ancestry score from genotyping; a clinical measure of PD severity; biopsy collection year; biopsy site on body; disease duration (years), and self-reported PD-related medication (A:amantadine, C:carbidopa, E:entacapone, L:levodopa, P:pramipexole, Ra:rasagiline, Ro:ropinirole, Rot:rotigotine, S:selegiline, T: trihexyphenidyl); cell expansion in 6-well ("6w") or from 12-well to 6-well format ("12w→6w"); date expanded cells were frozen; doubling time (days) during cell expansion. *: unconfirmed cell line, **: GBA mutation (see Methods). Colors within a column denote similar values.

File Name: Schiff-et-al.-Supplementary-Data.xlsx, Supplementary Data 2

Description: 5-fold Cross-validation strategy for healthy vs. PD classification. A subset of 74 cell lines from 74 individuals (6 *LRRK2* PD and paired controls, and 31 sporadic PD and paired controls was divided into 5 cross-validation folds. For each of 5 cross-validation sets, one fold of cell lines was held out in the test set. *: unconfirmed cell line (see Methods).
